# Data on mixing and curing methods effects on the compressive strength of concrete

**DOI:** 10.1016/j.dib.2018.03.095

**Published:** 2018-03-27

**Authors:** Ignatius O. Omuh, Timothy O. Mosaku, Opeyemi Joshua, Rapheal A. Ojelabi, Lekan M. Amusan, Adedeji O. Afolabi, Adeoluwa O. Arowolo

**Affiliations:** Department of Building Technology, Covenant University, Ota, Ogun state, Nigeria

## Abstract

Curing, though important is sometimes underrated in concrete production. This dataset provided shows the effects of four (4) different methods of curing on two distinct mix ratios. The data provided in this article are for a study that was conducted on one hundred and sixty (160) cube samples of mix ratios 1:2:4 and 1:1.5:3 while employing four (4) different methods of curing. The data given in the article displays the finding of the study. The findings can aid in prediction and optimization of concrete behavior and compressive strength when any of the curing methods are utilized.

**Specifications Table**

**Table:** 

Subject area	*Engineering, Concrete, Material Science Engineering Compressive Strength,Construction Site Practices*
More specific subject area	*Concrete, Cements, Compressive Strength*
Type of data	*Tables, graphs, and figure*
How data was acquired	*Laboratory simulation of site practices*
Data format	*Raw and analyzed*
Experimental factors	*One hundred and sixty (160) concrete cubes were produced simulating different mixing methods (manual and mechanical), different curing methods (immersion for 28 days, covering with impervious membrane for 28 days, immersion for 14 days and no curing at all)*
Experimental features	*There were two (2) mix ratios used- 1:2:4 and 1:1.5:3. The samples were tested for compressive strength after 28 days*
Data source location	*Ota, Ogun State, Nigeria*
Data accessibility	*The data is available within this article*

**Value of the data**

• This data is valuable because concrete is a widely used material and relevant information on its uses can be very significant.
• The data presented shows the effects of the different curing methods on the strength of concrete.
• The data may be relevant in the development of standards or codes of practice for concrete production in rural and arid areas.
• The data presented can be used to investigate the effects of mixing and curing methods on concrete of different mix proportions.
• The data presented can be used to develop an optimum method for concrete production.

## 1 Data

The data provided is focused on the effects of curing on the compressive strength of concrete. Concrete is a universally accepted construction material [1]. Majority of structures in Nigeria are built form concrete [2]. It's uses range from building elements like concrete beams, columns, slabs to bridges, piers, and roads. Unlike other construction materials, there is a variability in the strength of concrete. The final strength of concrete depends on several factors among which include the water binder ratio, the nature of the aggregates, the hydration rate of the concrete etc. [3]. Researches have been carried out on proper quality practice on construction sites and concrete production [4], [5], [6], [7], [8]. The results presented are from 160 cubes cast, cured, and tested for compressive strength.

## 2 Experimental design, materials and methods

**Fig. 1:**
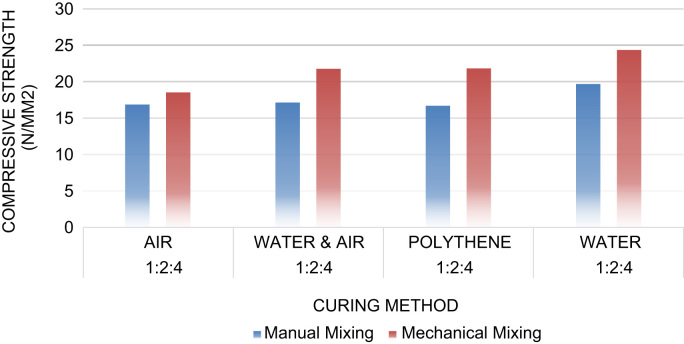
Compressive strength against curing methods for 1:2:4 concrete mix.

**Fig. 2:**
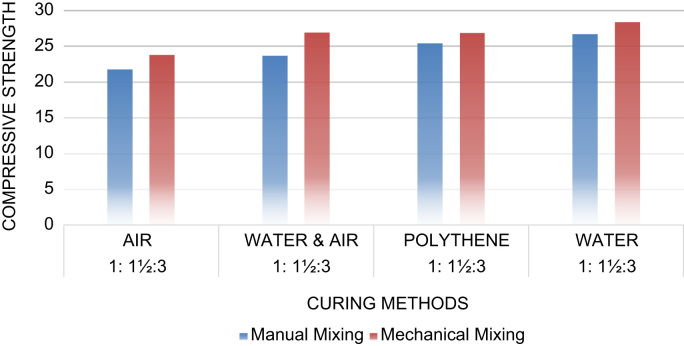
Compressive strength against curing methods for 1:1.5:3 concrete mix.

**Fig. 3:**
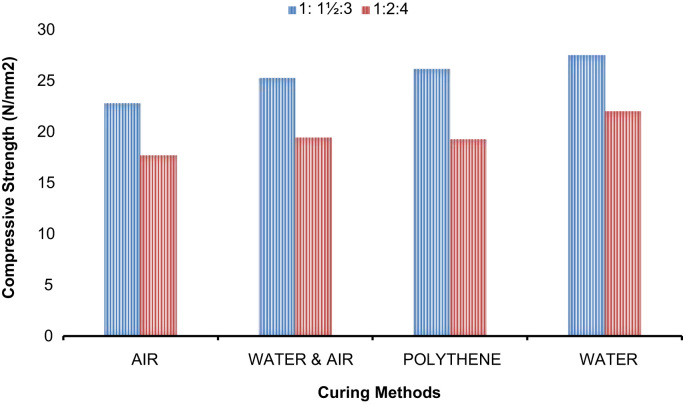
Compressive strength against curing methods.

**Fig. 4:**
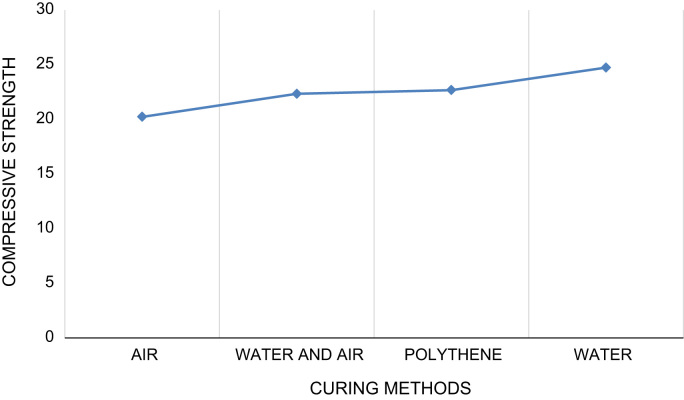
Average compressive strength against curing methods.

**Fig. 5:**
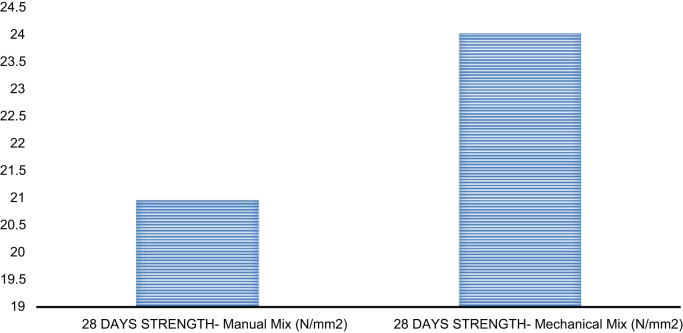
Compressive strength against mixing method.

The materials for this research were Ordinary Portland Cement (OPC) which was used as the binder throughout the research, fine, and coarse aggregates. The coarse aggregate size used 20 mm diameter, the fine aggregates used were graded appropriately. 160 concrete cube specimens were cast of dimensions 150 mm × 150 mm × 150 mm in steel mould in accordance to [9], [11]. Two different mix ratios were prepared according to [12], [13], [15], [18]. The cubes were demoulded after twenty-four hours and four different methods of curing were adopted in accordance to [10], [11], [17]. The first method was the immersion/ponding method. The second method was the membrane layer method. In this method, the concrete cubes were covered with polythene sheet and left airtight for a total of 28 days. The third method of curing was carried out by leaving the concrete cubes in the open and allowing air to act on the cubes, also for a total of 28 days. The final method of curing was achieved by immersing the cubes in water for 7 days and removing these cubes and allowing them to dry in the open for another 21 days before being tested for their various compressive strengths. At the conclusion of the hydration period of twenty-eight days, the concrete cube specimens were tested for compressive strength according to [9], [11], [14], [16]. This was achieved using a compression testing machine in the Building Technology laboratory in Covenant University (Fig. 1, Fig. 2, Fig. 3, Fig. 4, Fig. 5).
